# Non-fluorescent nanoscopic monitoring of a single trapped nanoparticle via nonlinear point sources

**DOI:** 10.1038/s41467-018-04689-5

**Published:** 2018-06-07

**Authors:** Seung Ju Yoon, Jungmin Lee, Sangyoon Han, Chang-Kyu Kim, Chi Won Ahn, Myung-Ki Kim, Yong-Hee Lee

**Affiliations:** 1 Department of Physics, Korea Advanced Institute of Science and Technology (KAIST), Daejeon, 34141 South Korea; 2 Department of Nano-Optical Engineering, Korea Polytechnic University, Siheung, 15073 South Korea; 3 KAIST, National NanoFab Center (NNFC), Daejeon, 341401 South Korea; 4 KU-KIST Graduate School of Converging Science and Technology, Korea University, Seoul, 02841 South Korea

## Abstract

Detection of single nanoparticles or molecules has often relied on fluorescent schemes. However, fluorescence detection approaches limit the range of investigable nanoparticles or molecules. Here, we propose and demonstrate a non-fluorescent nanoscopic trapping and monitoring platform that can trap a single sub-5-nm particle and monitor it with a pair of floating nonlinear point sources. The resonant photon funnelling into an extremely small volume of ~5 × 5 × 7 nm^3^ through the three-dimensionally tapered 5-nm-gap plasmonic nanoantenna enables the trapping of a 4-nm CdSe/ZnS quantum dot with low intensity of a 1560-nm continuous-wave laser, and the pumping of 1560-nm femtosecond laser pulses creates strong background-free second-harmonic point illumination sources at the two vertices of the nanoantenna. Under the stable trapping conditions, intermittent but intense nonlinear optical spikes are observed on top of the second-harmonic signal plateau, which is identified as the 3.0-Hz Kramers hopping of the quantum dot trapped in the 5-nm gap.

## Abstract (web summary)

Detection of single nanoparticles or molecules often relies on the attachment of fluorescent labels. Here, the authors demonstrate trapping a single nanoparticle on a bowtie nanoantenna and monitoring via second harmonic generation from the particle.

## Introduction

Over the past decades, the majority of optical studies for single nanoparticles and molecules have been performed by the fluorescence detection scheme, where the spatial resolution is less than 10 nm, breaking the diffraction limit.^1–6^ In the fluorescence detection scheme, the phosphor attached to the target substance acts as an isolated nano-light source, which allows the movement of the target substance to be traced in ultra-high spatial resolution. However, an additional pre-process of attaching the fluorescent material is required, and the attachment sometimes produces unpredictable changes in the intrinsic properties of the target substance. The availability of alternative non-fluorescent detection would greatly expand the range of investigable nanoparticles or molecules beyond species that are emissive and photostable, thus offering new applications in the fields other than molecular biophysics and imaging. There have been numerous attempts to detect a single nanoparticle or a single molecule without relying on fluorescence detection schemes, such as high-precision surface-enhanced Raman spectroscopy (SERS),^7,8^ cavity-enhanced detection methodology,^9^ interferometric scattering microscopy (iSCAT),^10^ centroid fitting methodology,^11^ and back-focal-plane interferometry.^12^

In recent years, plasmonically enhanced detection schemes using plasmonic nanoantennas, such as the circular double nanohole,^13^ nano-diabolo,^14^ nanorod,^9,15^ and bowtie^16^ plasmonic antennas, have actively been used for studies of single nanoparticles or molecules. The plasmonic nanoantenna can localize electromagnetic fields within a few nanometers, far beyond the diffraction limit, by utilizing surface-plasmon polaritons (SPPs), and thus strongly enhance the field intensity. Owing to the tight field concentration, the nanoantenna acts as a point-like illumination source that strongly increases the spatial resolution.^17–19^ Furthermore, the steep field gradient in the central region of the plasmonic nanoantenna generates an optical force strong enough to overcome the thermal Brownian fluctuations and to trap a single nanoparticle or a single molecule with low input power.^20,21^ In 2011, Pang et al. experimentally demonstrated the optical trapping of a single bovine serum albumin molecule with a 3.4-nm radius using a double nanoholes antenna.^22^ They observed single-molecule trapping events using transmission signals. However, in most conventional non-fluorescent plamsonic antenna-based schemes, a single wavelength light is used for both trapping and monitoring. This makes it difficult to distinguish the monitoring light from the trapping light and to control the trapping and monitoring processes independently.

In this work, we report the label-free nanoscopic monitoring of a single trapped nanoparticle by illuminating it with a pair of nonlinear point-like sources built in a three-dimensionally tapered plasmonic nanoantenna. A two-beam resonant pump system comprising a 1560-nm continuous-wave (CW) laser for trapping and a 1560-nm femtosecond (fs) laser for built-in second-harmonic (SH) illumination is employed for independent control of the trapping and monitoring processes. Resonant pumping into an extremely small volume of 5 × 5 × 7 nm^3^ through the tapered nanoantenna enables the low-power trapping of a nanoparticle and enhances nonlinear SH generation at the two vertices of the tapered nanoantenna. A pair of self-illuminating point-like nonlinear optical sources shines the quantum dot (QD) trapped in-between two potential wells formed in the nanoantenna. The Kramers hopping with a characteristic frequency of 3.0 Hz is identified by analysing the high-contrast nonlinear optical spikes.

## Results

### 3D plasmonic nanoantenna

**Fig. 1:**
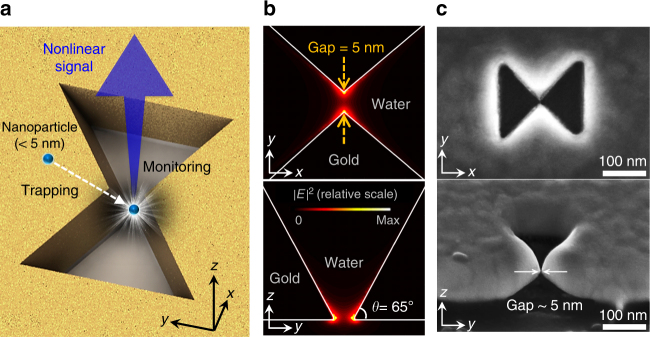
3D plasmonic nanoantenna. **a** Schematic of the proposed 3D plasmonic nanoantenna. Squeezed photons in an extremely small volume (*V*_m_ = 6.1 × 10^−8^*λ*^3^) of the 3D plasmonic nanoantenna enable the trapping of a sub-5-nm particle and create strong background-free second-harmonic signal. **b** |*E*|^2^ profiles of the antenna mode in the *xy*- and *yz*-plane. The resonant wavelength of the antenna is 1560 nm in water. The *y*-polarized incident beam is pumped from the bottom to excite the resonant mode. **c** Scanning electron microscopy images of the fabricated 3D plasmonic nanoantenna. The central-gap size (*g*) and the vertical taper angle are measured to be ~5 nm and ~65°, respectively

The proposed three-dimensional (3D) plasmonic nanoantenna platform is illustrated in Fig. 1a, in which a nanoscale air gap is introduced into a 100-nm-thick Au metal layer on a SiO_2_ substrate. It can be observed that the two vertices are tapered along all three dimensions. The minimum nanogap is formed at the center and bottom of the antenna, where the electric field is strongly concentrated.^23^ In the finite-difference time-domain (FDTD) simulation, the mode volume and field-intensity enhancement are calculated to be 6.1 × 10^−8^*λ*^3^ and 6.1 × 10^5^, respectively (see Supplementary Note 1). The length, width, and central gap size of the antenna are 200, 160, and 5.0 nm, respectively. The resonant wavelength (*λ*_ω_) of the antenna is 1560 nm in water. The incident laser (beam diameter = 2.0 μm) is pumped from the bottom. Figure 1b shows the calculated |*E*|^2^ profiles of the antenna mode in the *xy*- and *yz*-plane. Two maximum |*E*|^2^ points are notable at the apexes of the tapered nanoantenna. Here, the maximum electric fields are polarized perpendicular to the Au surface.^24^ Nonlinear SH signals (*λ*_2ω_ = 780 nm) are generated from the Au surface where the inversion symmetry is broken.^25^ In fact, the two maximum |*E*|^2^ points at the tapered vertices act as a pair of floating nonlinear point sources. It is worth emphasizing that *λ*_2ω_ is spectrally far enough from *λ*_ω_ such that the SH signal is background-free in principle. The 3D plasmonic nanoantenna is fabricated via proximal focused ion-milling (FIB) techniques on 100-nm-thick gold film deposited on a quartz substrate, as shown in Fig. 1c (see Supplementary Note 2). Here, the central-gap size and the vertical taper angle are measured to be ~5 nm and ~65°, respectively.

### Landscape of 3D optical potential well

**Fig. 2:**
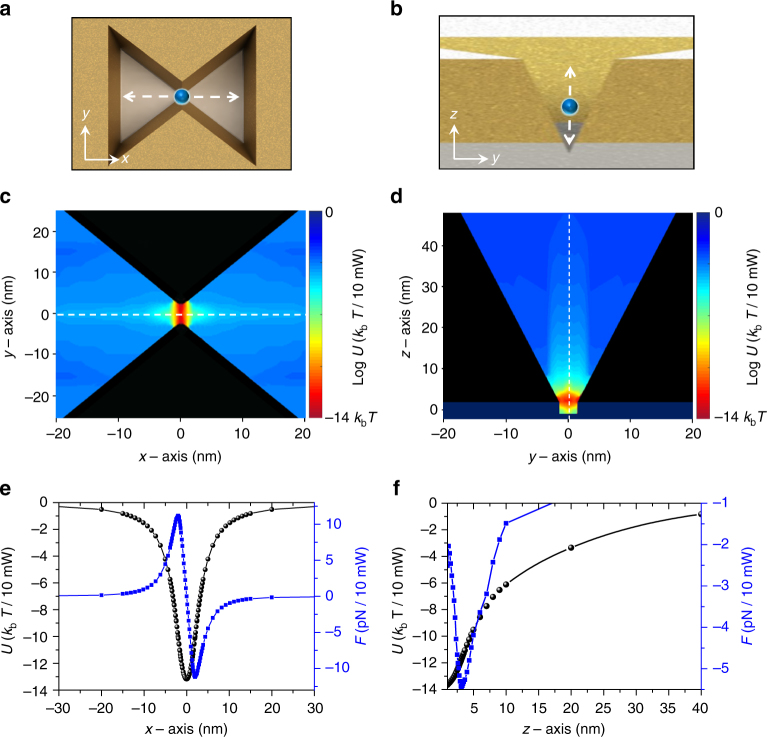
Optical potentials in 3D plasmonic nanoantenna. **a**, **b** Cross-sectional views of the plasmonic nanoantenna in the *xy*-plane and *yz*-plane, respectively. **c**, **d** Calculated optical potentials (*U*) in logarithm scale for a quantum dot (QD) moving along the *xy*-plane and *yz*-plane, respectively. Here, the incident pump power, central-gap size (*g*), and diameter of a QD are 10 mW, 5.0 nm, and 4.4 nm, respectively. **e**, **f** Optical potentials and the corresponding optical forces (*F*) along the *x*- and *z*-axis, respectively, near the center of the antenna

The optical trapping potential applied to a 4.4-nm CdSe/ZnS QD is numerically obtained in the proximity of the 3D plasmonic nanoantenna, by solving the Maxwell stress tensors (MSTs) in the FDTD simulation (see Methods). The calculated potential maps along the *xy*-plane at *z* = 0 and the *yz*-plane at *x* = 0 for an incident pump power of 10 mW are shown in Fig. 2a, c and 2b, d, respectively. Here, the central-gap size is fixed at 5.0 nm. The potential minimum is found at the center and bottom of the antenna, with a value of −14 *k*_b_*T*. Here, *k*_b_ is the Boltzmann constant, and *T* is the temperature (300 K). This potential of −14 *k*_b_*T* is deep enough to contain CdSe/ZnS QDs in the potential well.^14,15,26–28^ To obtain a better overview of the 3D potential landscape, we plot the potential and the corresponding optical force along the *x*- and *z*-axis near the center of the antenna, as shown in Fig. 2e, f, respectively. The maximum restoring force is found to be 11.2 pN when the QD is at *x* = ±2.0 nm. Along the *z*-direction, the optical force is largest at *z* = 3.2 nm. For antennas with larger central gaps of 8 and 10 nm, the corresponding potential depths are calculated to be −9.3 *k*_b_*T* and −2.0 *k*_b_*T*, respectively (see Supplementary Note 3).

**Fig. 3:**
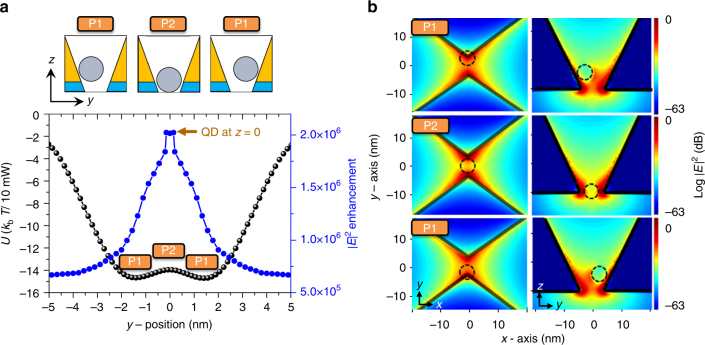
Double potential well in 3D plasmonic nanoantenna. **a** Optical potential (*U*) and |*E*|^2^ enhancement factor as a 4.4-nm quantum dot (QD) moves along the *y*-direction. A double potential well with two 14 *k*_b_*T*-deep wells and a central barrier of height 0.6 *k*_b_*T* is observed. Here, the *y*-position is defined as the projected position on the *y*-axis and the incident beam power is 10 mW. **b** |*E*|^2^ profiles in *xy*-planes and *yz*-planes when a QD is located at the positions of the potential well (P1) and the central barrier (P2). When a QD is placed at P2, the |*E*|^2^ enhancement is maximized to be 2.0 × 10^6^, which is 1.8 times larger than that of the QD at P1. This is because the low-refractive index region in the 5.0-nm gap is minimized when a QD is at P2

In the *yz*-plane potential profile, interesting features are observed. Figure 3a sketches an optical potential profile in the *yz*-plane for a 4.4-nm QD moving along the *y*-direction. The *y*-position is defined as the position of the QD projected onto the *y*-axis. We found two 14 *k*_b_*T*-deep potential wells with a central barrier of height 0.6 *k*_b_*T*. Note that two potential minima (*y* = ±1.5 nm) are located near the plasmonic hot points close to the apexes of the nanoantenna in Fig. 1b. The height 0.6 *k*_b_*T* of the central barrier is not large enough to stably hold a thermal QD in one of the two wells. Therefore, the QD can hop over the central barrier between two potential wells whose height tends to increase with the pump power (see Supplementary Note 4).

The electric-field profile also changes as the QD moves in the potential well, as shown in Fig. 3b. The solid blue dot in Fig. 3a represents the |*E*|^2^ enhancement factor with respect to the |*E*|^2^ of incident light. When the QD is placed at the bottom of the potential well (P1 position in Fig. 3, *y* = ±1.5 nm), the |*E*|^2^ enhancement is calculated to be 1.1 × 10^6^, which is 1.8 times larger than that obtained in the absence of a QD. When the QD is placed exactly on top of the potential barrier (P2 position in Fig. 3), the |*E*|^2^ enhancement is maximized to be 2.0 × 10^6^, which is 1.8 times larger than that of the QD at P1. This is because the low-refractive index region in the 5.0-nm gap is minimized when the QD is at P2, as shown in Fig. 3b. What we measure in the experiment is the nonlinear SH signals proportional to the value of |*E*|^4^ at the Au surface.^22,29,30^

### Experiment

**Fig. 4:**
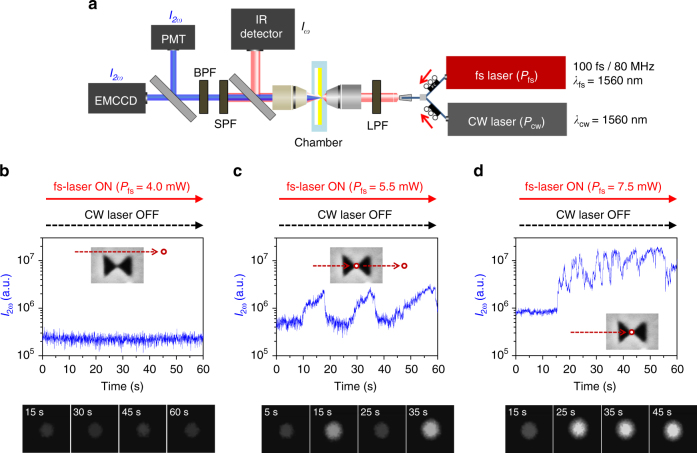
Experimental setup and monitoring of second-harmonic signals. **a** Two-laser scheme for simultaneous trapping and monitoring. A femtosecond (fs) pulse laser and a continuous-wave (CW) laser are independently used for monitoring and trapping, respectively. The two lasers are combined with a 50:50 coupler and the short wavelengths of the laser beam are filtered with a long-pass filter (LPF). The transmitted fundamental wave signal (*I*_ω_) is monitored by an infrared photodetector, and the short-wavelength second-harmonic (SH) signal (*I*_2ω_) filtered by a short-pass filter (SPF) and a band-pass filter (BPF) is monitored by an electron multiplying charge-coupled device (EMCCD) and a photomultiplier tube (PMT). **b**–**d** Time traces of SH intensities (*I*_2ω_) with different fs-laser powers (*P*_fs_ = 4.0, 5.5, and 7.5 mW) in the absence of the CW trapping laser. When *P*_fs_ = 4.0 mW, a stable nonlinear monitoring signal is observed

Armed with the theoretical study, we test our non-fluorescent trapping platform in a confocal optical setup [Fig. 4a, see Methods]. Two lasers—a fs pulse laser (100-fs pulses at 80 MHz) and a CW laser—are tuned to 1,560 nm for the resonant excitation of nanoantennas, for monitoring and trapping, respectively. The beam diameter focused through a 60× objective lens (numerical aperture = 0.65) is measured to be 2.0 μm, and the two lasers are polarized along the *y*-direction. The transmitted and reflected light is collected by photodetectors and an electron-multiplying charged-coupled device (EMCCD). CdSe/ZnS QDs (diameter ~4.0 nm, *λ*_emission_ ~620 nm) are dissolved in water with concentration of 2.0 × 10^−6^ M. Then they are injected into a chamber equipped with 3D nanoantennas. Note that the wavelengths of both pump beam (*λ*_ω_ = 1.56 μm) and SH signals (*λ*_2ω_ = 0.78 μm) are transparent to CdSe/ZnS QDs of interest.

First, the range of the fs-laser power *P*_fs_ suitable for nonlinear SH generation is investigated, in the absence of the CW trapping laser. With a low fs-laser power of 4.0 mW (intensity *I*_fs_ = *P*_fs_/*A*_beam_ = 0.12 MW cm^−2^, where *A*_beam_ is the area of the focused beam), stable nonlinear light *I*_2ω_ is observed, as shown in Fig. 4b. We take this baseline *I*_2ω_ as the background noise level at the monitoring wavelength of *λ*_2ω_. Here, the quietness of the baseline indicates the non-trapping of QD. When *P*_fs_ is increased to 5.5 mW (*I*_fs_ = 0.18 MW cm^−2^), as shown in Fig. 4c, the QD becomes trapped lightly and then escapes shortly in a random fashion. If *P*_fs_ is further increased to 7.5 mW (*I*_fs_ = 0.24 MW cm^−2^), the QD remains trapped, as shown in Fig. 4d. In all the subsequent experiments, the fs-laser power level is set to 4.0 mW to minimize the baseline *I*_2ω_.

**Fig. 5:**
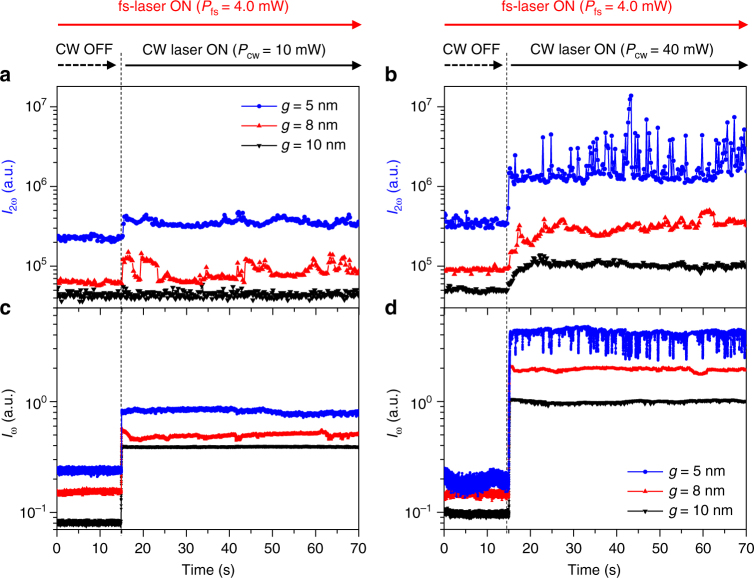
Simultaneous trapping and monitoring. **a**, **b** Time traces of second harmonic intensities (*I*_*2ω*_) and **c, d** transmitted fundamental wave intensities (*I*_ω_) with different CW laser powers of 10 and 40 mW, respectively, for three different nanoantennas with gap sizes (*g*) of 5, 8, and 10 nm. The CW laser is turned on at *t* = 15 s, and the 4.0-mW fs-laser remains on. The integration times for *I*_ω_ and *I*_2ω_ detection are 2 and 200 ms, respectively. Looking at the trapped moment (*t* = 15 s) of the quantum dot (QD), monitoring the *I*_ω_ signal makes it difficult to distinguish whether the QD is trapped, due to a sudden increase in the *I*_ω_ signal. On the other hand, the *I*_2ω_ signal clearly tells us whether the QD is trapped or not and even shows the dynamics of the trapped QD

The fs-laser and trapping CW laser are turned on at *t* = 0 and 15 s, respectively. In the regions of Fig. 5a, b where *t* < 15 s, we observe a clear dependence of the nonlinear background level on the gap size *g*, which is ascribed to the varying field strength.^23^ The relative heights of the background baselines are measured to be 5.1, 1.5, and 1.0, for *g* = 5, 8, and 10 nm, respectively. The corresponding relative transmittances of the fundamental wave (*λ*_ω_) are measured to be 2.9, 1.9, and 1.0, respectively, as shown in Fig. 5c, d. We also confirm that the pump CW laser produces a negligible SH background signal (see Supplementary Note 5).

When the CW laser is turned on (*t* = 15 s) in addition to the 4-mW fs-laser, the transmittance of the trapping CW laser *I*_ω_ at 1560 nm and the SH monitoring signal *I*_2ω_ at 780 nm exhibit rapid and dramatic changes. In our nanoscopy platform, two floating SH point sources at the vertices of nanoantenna automatically illuminate the QD once it is trapped between them. Because the SH signal has a quadratic dependence on the intensity, the *I*_2ω_ SH signal is expected to be a sensitive function of the QD position in the optical potential. That is, the signature of ultrafine motion of the QD in the 5-nm gap is recorded faithfully in the *I*_2ω_ signal, with a high signal-to-noise ratio. Looking at the trapped moment (*t* = 15 s) of the QD, monitoring the *I*_ω_ signal makes it difficult to distinguish whether the QD is trapped, due to a sudden increase in the *I*_ω_ signal when the trapping CW laser is turned on. On the other hand, the *I*_2ω_ signal clearly tells us whether the QD is trapped or not and even shows the dynamics of the trapped QD. The CW trapping laser power of 10 mW (*I*_cw_ = 0.32 MW cm^−2^) is found to be sufficient to trap a QD stably for the nanoantenna with a small gap of *g* = 5 nm but insufficient for antennas with a larger gap, as shown in Fig. 5.

When the trapping laser power *P*_cw_ is increased to 40 mW (*I*_cw_ = 1.27 MW cm^−2^), stable trapping is observed for all three antenna structures with *g* = 5, 8, and 10 nm. It is confirmed that a QD can be trapped in the antenna for more than 2 h. The trapping time, which is the time required for a QD to reach a stable trapped state, shows a noticeable gap-size dependence. It is measured as 0.5, 2.8, and 6.4 s for *g* = 5, 8, and 10 nm, respectively. With a 4-mW fs-laser, the average SH intensities normalized to the nonlinear background level are measured to be 5.6, 3.3, and 2.1, for *g* = 5, 8, and 10 nm, respectively. Once the CW laser is turned off, the QD escapes the antenna immediately (see Supplementary Note 5).

## Discussion

**Fig. 6:**
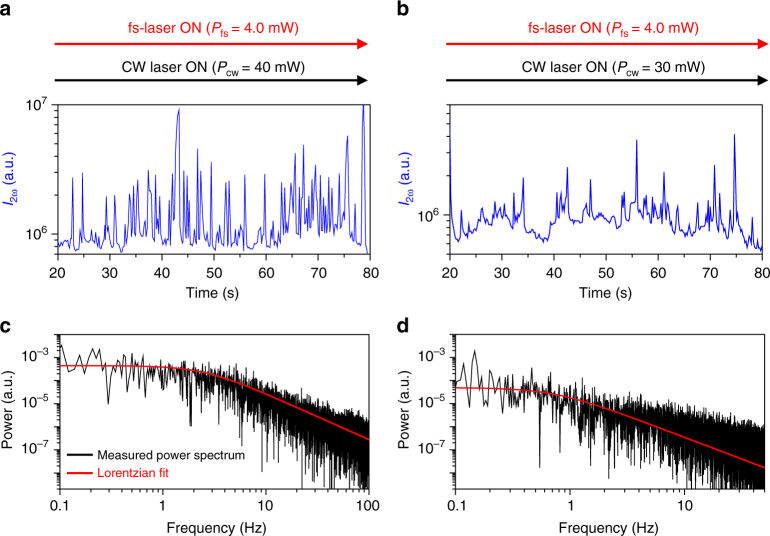
Kramers hopping. **a**, **b** Measured second harmonic (SH) spike signals (*I*_2ω_) with trapping CW laser powers *P*_cw_ of 40 and 30 mW, respectively, where the 4.0-mW fs-laser is on. These signals are estimated to be generated by the low frequency Kramers hopping associated with the double potential well formed near the center of the nanoantenna. **c**, **d** Power spectra (black lines) of the measured SH signals and the Lorentzian fitting curves (red lines). When *P*_cw_ is 40 and 30 mW, the roll-off frequency is measured as 3.0 and 0.5 Hz, respectively

A series of intense SH spikes are observed from a trapped 4-nm CdSe/ZnS QD when the 3D tapered nanoantenna is resonantly pumped at a wavelength of 1560 nm with a 40-mW CW laser and a 4-mW fs-laser, as shown in Fig. 5b. Additionally, in the transmitted fundamental signal (*I*_ω_), similar negative spikes are simultaneously observed mainly due to the translational movement of the trapped QD, as shown in Fig. 5d (see Supplementary Note 5). We attribute this phenomenon to the Kramers hopping associated with the double potential well formed near the two hot plasmonic points discussed previously, as shown in Fig. 3. The low-frequency characteristics of the SH spikes supports our interpretation.^19^ That is, the QD hops back and forth between two shallow potential wells (P1 positions in Fig. 3) randomly at a low frequency. It generates a strong SH signal spike at the instant that the QD passes by the P2 position in Fig. 3. When the QD is located at P2, the field intensity at the antenna gap is calculated to be 1.8 times larger than that at the bottom of the well (P1), as shown in Fig. 3. Dielectric loading of the nano-metallic gap with a trapped QD is found to enhance the electric field and surface current density responsible for the SH generation. The SH signal intensity with a QD at P2 is expected to increase by a factor of 1.8^2^ (=3.24) with respect to that with a QD at P1. This ratio agrees with the average intensity increment (3.26 times) of the intermittent SH spikes shown in Fig. 6a. The hopping rate is analysed by using the power spectrum density method, as shown in Fig. 6c. The roll-off frequency (corner frequency) of the Kramers hopping is estimated to be 3.0 Hz. In additional experiments, we found that the roll-off frequency decreases with decreasing *P*_cw_ (see Supplementary Note 6). This is understandable considering that the height of the potential barrier decreases with decreasing *P*_cw_. For example, when *P*_cw_ = 30 mW, the roll-off frequency is measured as 0.5 Hz, as shown in Fig. 6b, d. This is the unambiguous observation that a single nanoparticle trapped in the plasmonic nanoantenna exhibits Kramers hopping.

In summary, we have demonstrated the non-fluorescent nanoscopic monitoring of a single trapped nanoparticle by illuminating it with a pair of floating nonlinear point sources built in a plasmonic nanoantenna. A two-beam resonant pump system comprising a 1560-nm CW laser for trapping and a fs*-*laser for built-in SH illumination is employed for independent control of the trapping and monitoring processes. The resonant pumping into an extremely small mode volume of 5 × 5 × 7 nm^3^ enables the trapping and monitoring of a 4-nm CdSe/ZnS QD with low optical intensity less than 0.4 MW cm^−2^. Intense nonlinear optical spikes are observed when a 4-nm CdSe/ZnS QD crosses the potential barrier in the middle of a symmetric double potential well formed between two apexes of the three-dimensionally tapered plasmonic nanoantenna with 5-nm gap. The Kramers hopping of the 3.0-Hz characteristic frequency is identified by analysing the high-contrast nonlinear optical spikes. The label-free and high-contrast characteristics of the proposed scheme are believed to be advantageous for future quantum and bio-optical research.

## Methods

### Optical potential and force calculations

The electromagnetic force applied to a particle by harmonic fields can be calculated using the Maxwell stress tensor (MST). We calculate the electromagnetic field around the particle in an FDTD simulation and apply the MST method. The simulations are conducted numerically using a commercially available FDTD software package (Lumerical Solutions, Inc.; https://www.lumerical.com/).

The Lorentz force per unit volume is

1 $${\mathbf{f}} = \rho {\mathbf{E}} + \,{\mathbf{J}} \times {\mathbf{E}},$$

where *ρ* is the total charge per unit volume, and **J** is the total current density.

According to Maxwell’s equations,

2 $$\rho = \varepsilon \nabla \cdot {\mathbf{E}},{\mathbf{J}} = \frac{1}{{{\mu }}}\nabla \times {\mathbf{B}} - {\mathrm{\varepsilon }}\frac{{\partial {\mathbf{E}}}}{{\partial t}},$$

and according to the product rule,

3 $$\frac{\partial }{{\partial t}}\left( {{\mathbf{E}} \times {\mathbf{B}}} \right) = \frac{{\partial {\mathbf{E}}}}{{\partial t}} \times {\mathbf{B}} + {\mathbf{E}} \times \frac{{\partial {\mathbf{B}}}}{{\partial t}} \cdot$$

Eq. (1) then becomes

4 $${\mathbf{f}} = \varepsilon \left[ {\left( {\nabla \cdot {\mathbf{E}}} \right){\mathbf{E}} - {\mathbf{E}} \times \left( {\nabla \times {\mathbf{E}}} \right)} \right] + \frac{1}{\mu }\left[ {\left( {\nabla \cdot {\mathbf{B}}} \right){\mathbf{B}} - {\mathbf{B}} \times \left( {\nabla \times {\mathbf{B}}} \right)} \right] - \varepsilon \frac{\partial }{{\partial t}}\left( {{\mathbf{E}} \times {\mathbf{B}}} \right) \cdot$$

After eliminating the curls, using the vector calculus identity, we obtain

5 $$\nabla \left( {{\mathbf{A}} \cdot {\mathbf{B}}} \right) = \left( {{\mathbf{A}} \cdot \nabla } \right){\mathbf{B}} + \left( {{\mathbf{B}} \cdot \nabla } \right){\mathbf{A}} + {\mathbf{A}} \times \left( {\nabla \times {\mathbf{B}}} \right) + {\mathbf{B}} \times \left( {\nabla \times {\mathbf{A}}} \right) \cdot$$

With the Poynting vector ${\mathbf{S}} = \frac{1}{{{\mu }}}{\mathbf{E}} \times {\mathbf{B}}$, Eq. (4) is expressed as

6 $$\begin{array}{*{20}{c}} {\mathbf{f}} & = & \hskip -3pc {\varepsilon \left[ {\left( {\nabla \cdot {\mathbf{E}}} \right){\mathbf{E}} - \left( {{\mathbf{E}} \cdot \nabla } \right){\mathbf{E}} - \frac{1}{2}\nabla E^2} \right]} \\ {} & + & {\frac{1}{{{\mu }}}\left[ {\left( {\nabla \cdot {\mathbf{B}}} \right){\mathbf{B}} - \left( {{\mathbf{B}} \cdot \nabla } \right){\mathbf{B}} - \frac{1}{2}\nabla B^2} \right] - \varepsilon \mu \frac{{\partial {\mathbf{S}}}}{{\partial t}} \cdot } \end{array}$$

Now, the MST $\overline T$ is a 3 × 3 matrix with components defined by

7 $$\overline T _{ij} = \varepsilon \left( {{\mathrm{E}}_i{\mathrm{E}}_j - \frac{1}{2}{\mathrm{\delta }}_{ij}E^2} \right) + \frac{1}{\mu }\left( {{\mathrm{B}}_i{\mathrm{B}}_j - \frac{1}{2}{\mathrm{\delta }}_{ij}B^2} \right) \cdot$$

The last term in Eq. (6) is eliminated via time averaging. As a result, the time-averaged force is simplified as

8 $$\left\langle {\mathbf{f}} \right\rangle _{{\mathrm{time}} - {\mathrm{averaged}}} =\left\langle{ \nabla \cdot {\mathbf{T}}}\right\rangle_{{\mathrm{time}} - {\mathrm{averaged}}} .$$

Thus, the total force on the particle is obtained as the volume integral of the particle.

Using the relationship between the force and the potential, we obtain

9 $${\mathbf{U}} = - {\int}_{{\mathbf{ref}}}^{\boldsymbol{r}} {{\vec{\mathbf F}} \cdot \mathrm{d}{\vec{\mathbf r}}.}$$

Thus, the optical potential is calculated. The potential at infinity is considered to be zero.

In the FDTD simulations, we compute the electromagnetic field numerically when the 4.4 nm size particle is in each position in the cavity. The grid size of FDTD calculation is 0.5 nm × 0.5 nm × 0.5 nm. The nanoantenna dimensions are considered to be 200 × 160 × 100 nm in length, width, and thickness, respectively, and the 3D taper angle is 65°.

### Optical measurements

We use the confocal optical setup and control the trapping event time by separating the probe laser and trapping laser, as shown in Fig. 4a. A femtosecond pulse laser (Toptica FemtoFiber pro NIR, *λ* = 1.56 µm, 100-fs pulse, 80 MHz) with a peak power of approximately 0.5 kW is employed as a probe laser for generating the SH signal and illuminates the cavity with an incident power of 0.12 MW cm^−2^ during the optical trapping. A CW laser (Thorlab, Product No. FPL1009S, *λ* = 1.56 µm) is additionally introduced into the cavity with an incident power from 0.32 up to 1.27 MW cm^−2^ and independently controls trapping events as a trapping laser. The two beams are combined through a 50:50 beam coupler and focused onto the sample through the microscope objective (60×, 0.65 NA) with transverse direction polarization (*y*-axis in Fig. 1). The transmitted fundamental beam is detected by an infrared photodiode (Femtowatt InGaAs Photo-receiver, Newport, model:2153). Simultaneously, the SH signal is detected by the visible EMCCD (Andor technology, iXon3 897) and visible photomultiplier tube (Princeton Instruments, PD-471) after passing through both the short-pass filter (SPF, *λ*_cut-off_ = 1300 nm) and the bandpass filter (BPF, *λ*_center_ = 780 ± 10 nm). The time traces of the SH and fundamental signal are recorded simultaneously in real time. Prior to the optical trapping experiments, the nanoantenna is integrated in the microfluidic chamber with QDs dissolved in water with concentration of 2 × 10^−6^ M. The QD (Nanosquare, Product No. C01-SH01-AC-100620, standard emission peak = 620 nm) is CdSe/ZnS (core/shell type) and 4.4 nm in diameter, as specified by the manufacturer.

### Data availability

The data sets within the article and Supplementary Information of the current study are available from the authors upon request.

## Electronic supplementary material

Supplementary Information

Peer Review Report
