# Supplementary material for: Non-fluorescent nanoscopic monitoring of a single trapped nanoparticle via nonlinear point sources
Source: Nat Commun. 2018 Jun 7;9:2218. doi: 10.1038/s41467-018-04689-5 (PMC5992221; doi:10.1038/s41467-018-04689-5)
Supplement: Supplementary file 1 — Supplementary Information [file 41467_2018_4689_MOESM1_ESM.pdf]

Supplementary Information for

Non-fluorescent nanoscopic monitoring of a single trapped nanoparticle via nonlinear point sources

Yoon *et al.*

Supplementary Note 1: Field enhancement in plasmonic nanoantenna. The mode volume and $|E|^2$ enhancement of the 3D gap-plasmon antenna are plotted as a function of g in Supplementary Fig. 1. Here, the vertical angle (θ) is fixed at 65°, and the length and width of the antenna are 200 and 160 nm, respectively. The pump beam (beam diameter = 2.0 μm) is illuminated from the SiO_2 substrate side. The $|E|^2$ enhancement is defined as the ratio between the maximum $|E|^2$ values of the antenna mode and the incident wave. Photon confinement is clearly noticeable when g is less than 20 nm. When g is reduced from 50 nm to 2 nm, the mode volume decreases from $4.0 \times 10^{-5}\ \lambda^3$ to $4.4 \times 10^{-9}\ \lambda^3$ and the $|E|^2$ enhancement increases from 1.5×10^4 to 2.0×10^6. For an antenna with g = 5 nm, the mode volume and $|E|^2$ enhancement are calculated to be $6.1 \times 10^{-8}\ \lambda^3$ and 6.1×10^5, respectively. The $|E|^2$ enhancement is dependent on the mode volume, coupling efficiency, and joule losses. Here, the coupling efficiency is estimated to be 20 %, and the ratio of the metal absorption among the total loss from the antenna is 25 %.

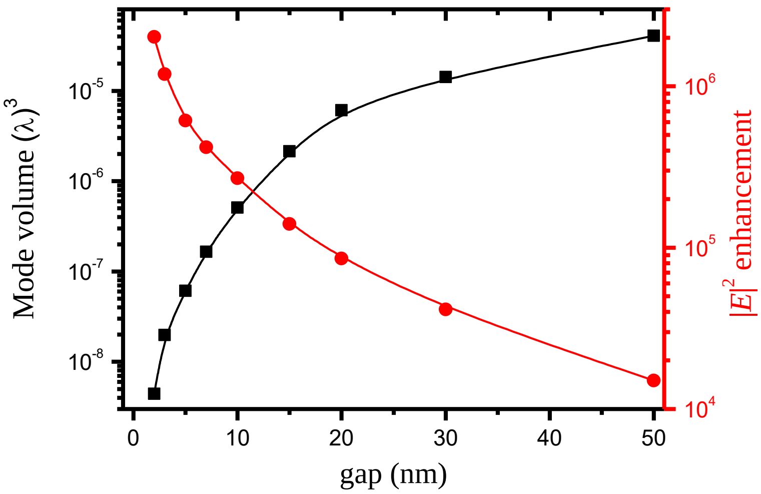

Supplementary Figure 1. Field enhancement in 3D plasmonic nanoantenna. Mode volume (black curve) and $|E|^2$ enhancement (red curve) as a function of g at a fixed vertical angle (θ) of 65°. When g = 5 nm, the mode volume and $|E|^2$ enhancement are calculated to be $6.1 \times 10^{-8}\ \lambda^3$ and 6.1×10^5, respectively. Here, all the simulation data were taken at resonant wavelength.

Supplementary Note 2: Fabrication of 3D plasmonic nanoantenna. To fabricate the 3D plasmonic nanoantennas, we applied proximal milling techniques in Ga^{+}-based focused ion beam (FIB) processes (model: Helios NanoLab FEI) to a 100-nm-thick evaporated gold film on a fused silica coverslip. We intentionally off-designed the milling patterns from the original antenna design to utilize the proximity effect of FIB milling. By optimally controlling the distance (s) between two triangular milling patterns, as shown in Supplementary Fig. 2a, we overcame the FIB resolution limit (~10 nm) and successfully fabricated the sub-5-nm-gap 3D plasmonic nanoantenna, as shown in Supplementary Fig. 2b. The vertical taper angle (θ) was created by the proximity effect during the FIB milling, as shown in Supplementary Fig. 2c. θ is measured to be ~65°. A 100-nm-thick gold film was sputtered on a fused quartz substrate. The purity of the Au used for evaporation exceeded 99.99%. The evaporation rate was 0.5 Å per second, and the root-mean-square and peak-to-peak roughness of the deposited gold film were 0.8 and 5 nm, respectively, as ascertained via atomic force microscopy.

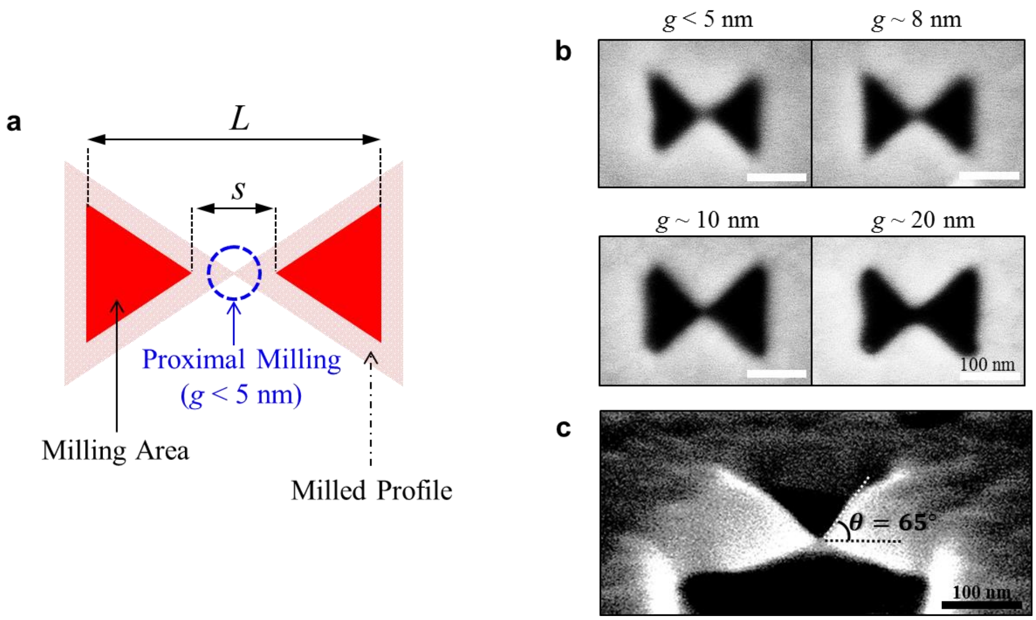

Supplementary Figure 2. Fabrication of 3D plasmonic nanoantenna. a Proximal milling technique in the FIB process. **b** Top views of scanning electron microscopy images of the fabricated 3D plasmonic nanoantennas with different milling parameters of s and L. Scale bars = 100 nm. **c** 45°-tilted view of the fabricated 3D plasmonic nanoantenna.

Supplementary Note 3: Optical potentials with different antenna gaps. In nanoantennas with different gap sizes (*g*) of 5, 8, 10, 14, and 20 nm, the optical potentials and forces felt by a 4.4-nm CdSe/ZnS quantum dot (QD) moving along the *z*-axis are numerically calculated in Supplementary Fig. 3. The potential well depth increases as *g* decreases, as shown in Supplementary Fig. 3a. The potential minima are calculated to be -14 k_bT, -9 k_bT, and -2 k_bT for antennas with *g* values of 5, 8, and 10 nm, respectively. Here, the 10-mW incident light (beam diameter = 2.0 μm) is pumped from the SiO_2-substrate side. The corresponding optical forces are plotted in Supplementary Fig. 3b, where the maximum restoring forces are calculated to be 5.5, 4.3, and 0.6 pN at *z* = 3, 5, and 6 nm for *g* values of 5, 8, and 10 nm, respectively. When *g* > 14 nm, the potential depth and restoring force are reduced to < 0.4 k_bT and < 0.2 pN, respectively.

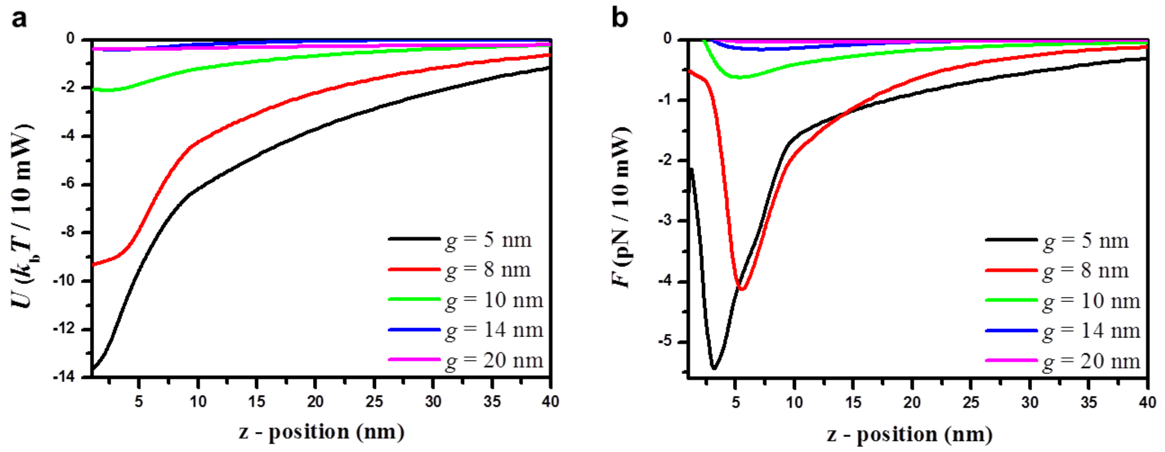

Supplementary Figure 3. Optical potential and force with different antenna gap. **a**, **b** Calculated optical potentials (*U*) and optical forces (*F*), respectively, for the case where a quantum dot (QD) is moved on the *z*-axis along the centre of the antenna with different central-gap sizes (*g*).

Supplementary Note 4: Optical potentials with different input powers. Supplementary Fig. 4 shows the formation of the double potential well for a 4.4-nm QD moving along the *y*-direction with different input powers. The depth of the two-potential well increases to 14.6 $k_b T$, 29.2 $k_b T$, 43.8 $k_b T$, and 58.4 $k_b T$ as the pumping power is increased to 10, 20, 30, and 40 mW, respectively. Simultaneously, the central barrier potential increases to 0.6 $k_b T$, 1.2 $k_b T$, 1.8 $k_b T$, and 2.4 $k_b T$, respectively. Two potential minima ($y = \pm 1.5$ nm) are located near the plasmonic hot points close to the apexes of the nanoantenna, as shown in Fig. 1b of the main text. The QD can hop over the central barrier between the two-potential well, and the hopping rate depends on the ratio of the potential-well depth to the height of the central barrier [1–3].

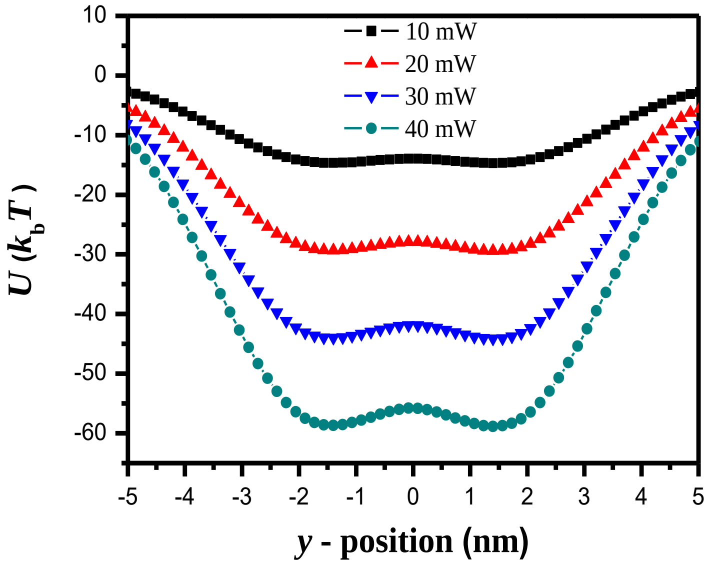

Supplementary Figure 4. Optical potential with different input power. Calculated optical potentials (*U*) with different input powers. Here, a quantum dot is moved along the *y*-direction from the gap centre.

Supplementary Note 5: Monitoring without/with QDs. We verify that the second-harmonic (SH) generation is not affected by the trapping CW laser in the absence of the QDs. Supplementary Fig. 5 shows the time traces of the fundamental wave intensity (I_ω) and the SH signal intensity ($I_{2\omega}$) for the case where a 40-mW trapping CW laser is turned on (t = 26 s) and off (t = 156 s) under the 4-mW monitoring femtosecond (fs) laser pumping. The background base SH signal is observed when the trapping CW laser is off. This SH signal maintains the base power even after the trapping laser is turned on. Thus, the trapping CW laser with a power of < 40 mW produces a negligible SH background signal. That is, the time-tracing SH-signal ($I_{2\omega}$) with QDs purely represents the dynamics of the QD in the 5-nm gap of the antenna, as shown in Supplementary Fig. 6. Once the CW laser is turned off, the QD escapes the antenna immediately. Supplementary Fig. 7 shows the control experiment result when the trapping CW laser is turned on and off while the detection fs-laser is off.

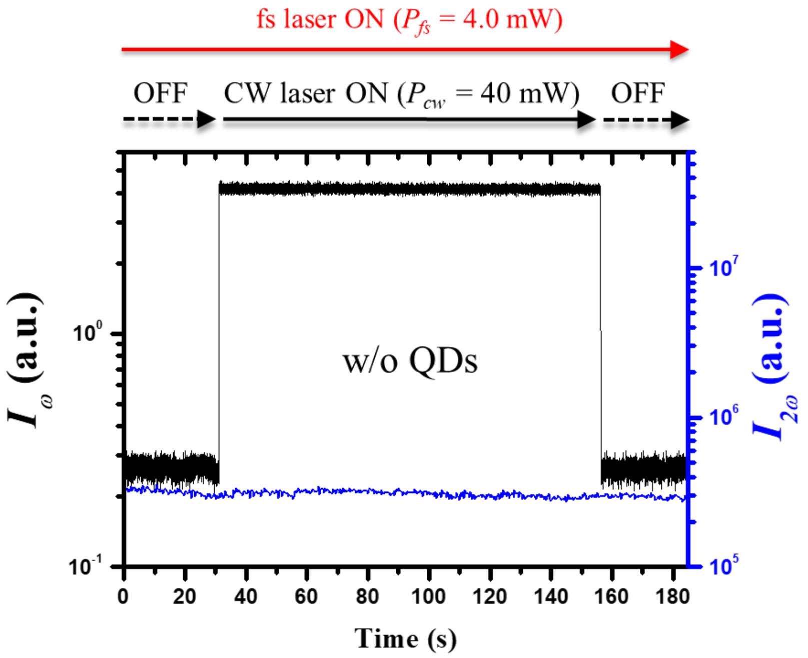

Supplementary Figure 5. Monitoring without QDs. Time traces of the measured second-harmonic signal intensity ($I_{2\omega}$) and transmitted fundamental-wave intensity (I_ω) without QDs. Here, the CW laser (P_{cw} = 40 mW) is turned on (t = 30 s) and off (t = 155 s) at a fixed femtosecond laser power (P_{fs}) of 4.0 mW.

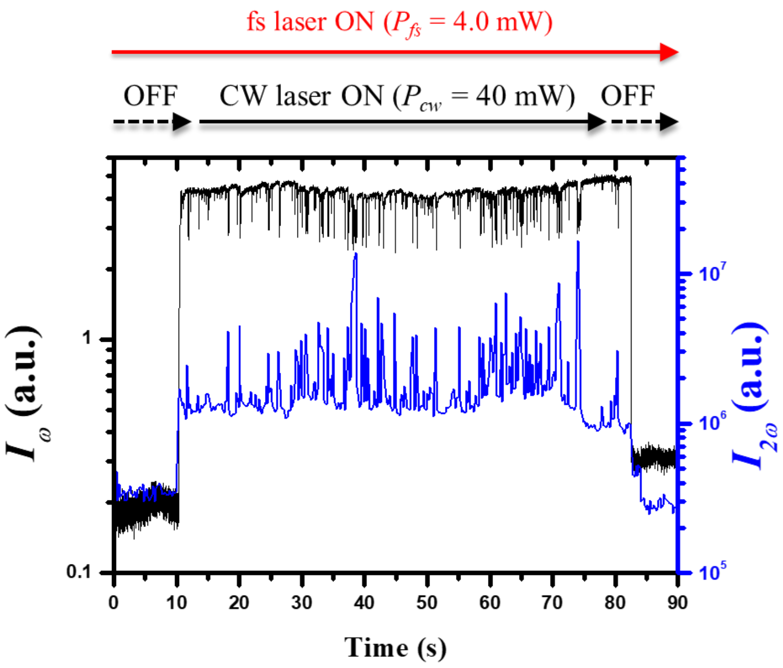

Supplementary Figure 6. Monitoring with QDs. Time traces of the measured SH-signal intensity ($I_{2\omega}$) and transmitted fundamental-signal intensity (I_ω) with QDs. Here, the CW laser (P_{cw} = 40 mW) is turned on (t = 10 s) and off (t = 84 s) at a fixed femtosecond laser power (P_{fs}) of 4.0 mW.

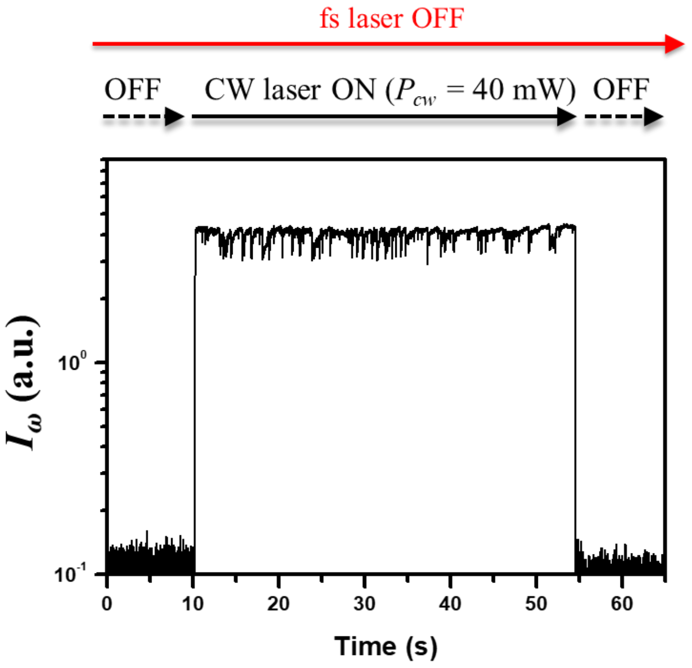

Supplementary Figure 7. Monitoring with QDs. Time trace of the measured transmitted I_ω intensity with QDs. Here, the CW laser (P_{cw} = 40 mW) is turned on (t = 10 s) and off (t = 55 s) while the fs-laser is turned off.

Supplementary Note 6: Kramers Hopping. The fundamental (I_ω) and second-harmonic ($I_{2\omega}$) spike signals are observed when a 40-mW CW laser and a 4-mW fs-laser are pumped, as shown in Supplementary Fig. 8a. And, both signals are synchronized with each other, indicating that the $I_{2\omega}$ fluctuation is mainly due to the translational movement of the trapped QD. We attribute this phenomenon to the Kramers hopping associated with the double potential well formed near the two hot plasmonic points, as shown in Supplementary Fig. 8b. The low-frequency characteristics of these spikes support our interpretation. That is, the QD hops back and forth between two shallow potential wells randomly at a low frequency. It generates negative I_ω and positive $I_{2\omega}$ spikes at the instant that the QD passes by the barrier, due to the sudden decrease of transmittance and increase of $|E|^2$ enhancement, respectively, as shown in Supplementary Fig. 8b.

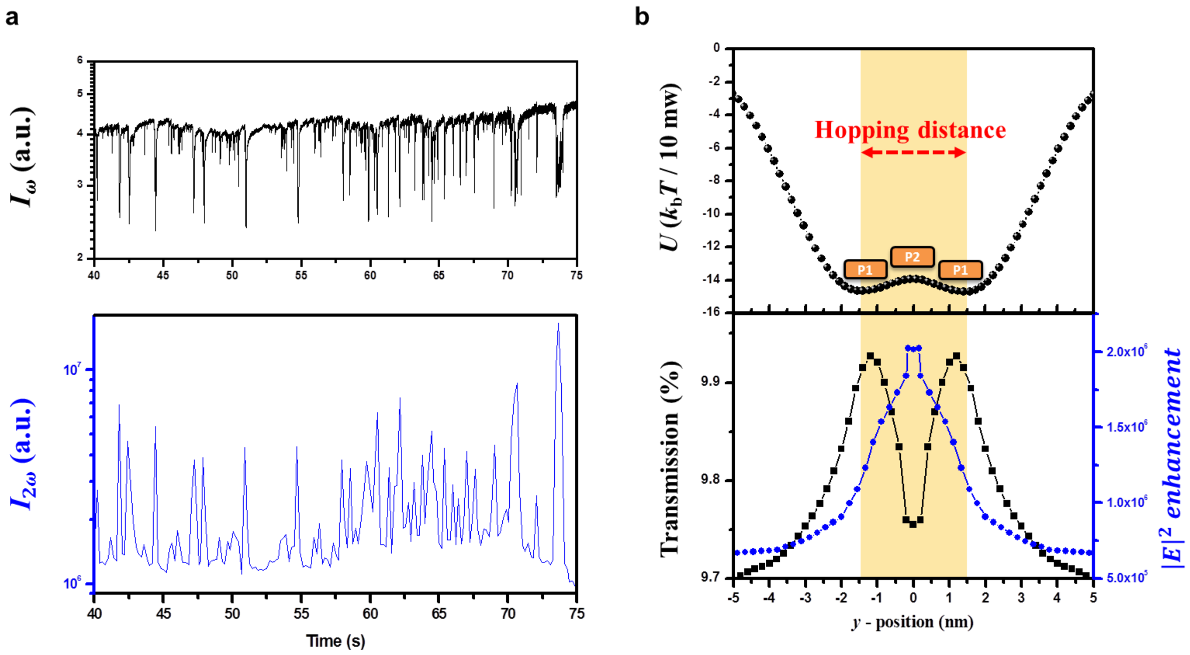

Supplementary Figure 8. Kramers hopping. a Measured fundamental (I_ω) and second-harmonic ($I_{2\omega}$) signals under the condition of P_{cw} = 40 mW and P_{fs} = 4.0 mW. Here, the integration times for I_ω and $I_{2\omega}$ detection are 2 ms and 200 ms, respectively. **b** Calculated optical potential, transmission, and $|E|^2$ enhancement when a 4.4-nm QD moves along the y-direction.

The hopping rate is analysed by using the power spectral density (PSD) method, as shown in Figs. 6c and 6d of the main text. The roll-off frequency (corner frequency) of the Kramers hopping is estimated to be 3.0 and 0.5 Hz for P_{cw} of 40 and 30 mW, respectively. In order to clarify that our PSD data supports the "Krammrs hopping" instead of a "diffusion in a harmonic potential", we estimate the roll-off frequency of oscillation, assuming that a QD is trapped in a single harmonic potential [4 - 7]. In this case, the roll-off frequency is given by $f_0 = \alpha(2\pi\beta)^{-1}$, where α is the trap stiffness, β is the drag coefficient ($\beta = 6\pi\eta r$), r is the particle radius, and η is the viscosity of the water. And, the trapping stiffness α can be calculated using the equipartition principle of $\alpha < x^2 >= k_{\mathrm{b}}T$. Here, we set x = 5 nm, because the potential width is comparable to the size of the antenna gap. Using those parameters, the roll-off frequency is estimated to be $f_0 \sim 6 \times 10^5$ Hz, which is significantly larger than the frequency (a few Hz) obtained from our experiment. So, the assumption that the particle oscillates simply in a single harmonic potential well can be ruled out.

We additionally tested the power dependence on the roll-off frequency, as shown in Supplementary Fig. 9, as increasing the trapping CW laser power (P_{cw}) from 10 to 40 mW. We can see here that the roll-off frequency increases with the pumping power, where the roll-off frequency is in the range of 0 ~ 3 Hz. This observation indicates that the trapped QD is in the underdamped state of the Kramers hopping [8 - 10].

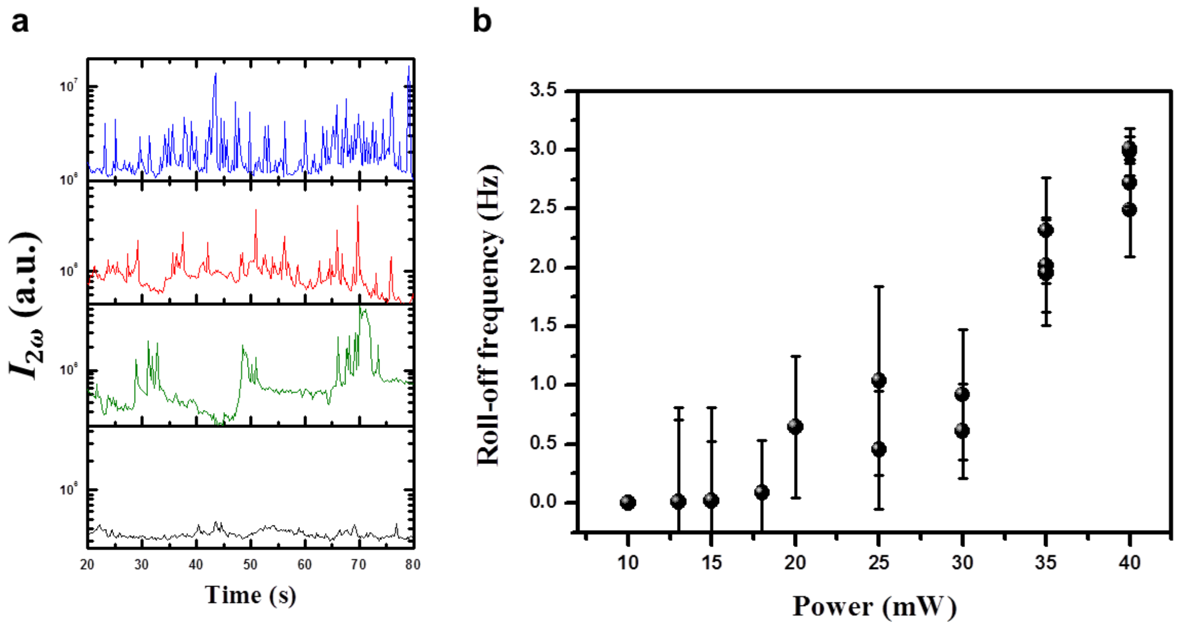

Supplementary Figure 9. Kramers hopping with different input power. a Measured SH signals ($I_{2\omega}$) with different CW laser powers (P_{cw}) of 40 mW(blue line), 30 mW(red line), 20 mW(green line) and 10 mW (black line). **b** Measured roll-off frequency as a function of P_{cw}. Here, the error bar represents the standard deviation of the Lorentzian fitting error.

Supplementary References

[1] McCann L. I., Dykman M. & Golding B. Thermally activated transitions in a bistable three-dimensional optical trap. *Nature* **402,** 785 (1999)

[2] Wilking, J. N. & Mason, T. G. Multiple trapped states and angular Kramers hopping of complex dielectric shapes in a simple optical trap. *Europhys. Lett.* **81,** 58005 (2008)

[3] Zehtabi-Oskuie, A., Jiang, H., Cyr, B. R., Rennehan, D. W., Al-Balushia, A. A. & Gordon, R. Double nanohole optical trapping: dynamics and protein-antibody co-trapping. *Lab Chip.* **13,** 2563 (2013)

[4] Mestres, P., Berthelot, J., Aćimović, S. S & Quidant, R. Unraveling the optomechanical nature of plasmonic trapping. *Light Sci. Appl.* **5**, 16092 (2016)

[5] Lee, W. M., Reece, P. J, Marchington, R. F, Metzger, N. K & Dholakia, K. Construction and calibration of an optical trap on a fluorescence optical microscope. *Nat. Protoc.* **2**, 3226 - 3238 (2007)

[6] van der Horst, A. & Forde, N. R. Power spectral analysis for optical trap stiffness calibration from high-speed camera position detection with limited bandwidth. *Opt. Express* **18**, 7670-7677 (2010)

[7] Salakhutdinov, V. *et al.* Optical trapping of nanoparticles by full solid-angle focusing. *Optica* **3**, 1181-1186 (2016)

[8] Kramers, H. A. Brownian motion in a field of force and the diffusion model of chemical reactions. Physica **7**, 4, 284-304 (1940)

[9] Mel'nikov, V. I. The Kramers problem: Fifty years of development. *Phys. Rep*. **209**, 1-71 (1991)

[10] Rondin, L. *et al.* Direct measurement of Kramers turnover with a levitated nanoparticle. *Nat. Nanotechnol*. **12**, 1130–1133 (2017)
