# Supplementary material for: Non-fluorescent nanoscopic monitoring of a single trapped nanoparticle via nonlinear point sources
Source: Nat Commun. 2018 Jun 7;9:2218. doi: 10.1038/s41467-018-04689-5 (PMC5992221; doi:10.1038/s41467-018-04689-5)
Supplement: Supplementary file 2 — Peer Review Report [file 41467_2018_4689_MOESM2_ESM.pdf]

Reviewers' comments:

Reviewer #1 (Remarks to the Author):

In the manuscript "Non-fluorescent nanoscopic monitoring of a single trapped nanoparticle via nonlinear point sources" the authors use CW and fs lasers to trap quantum dots with nanoapertures in gold film. They monitor the trapping by changes to the fundamental and second harmonic. The second harmonic shows greater sensitivity to nearfield intensity and therefore, they suggest that the fluctuations observed are from Kramers hopping. Overall, I think that the manuscript presents remarkable trapping results with substantial SHG from a single 4 nm QD. I am not entirely convinced of the Kramers hopping hypothesis, but I do not feel this should preclude publication.

1) I note that the authors have not carefully reviewed the literature. For example, there is a work on trapping of quantum dots and two photon fluorescence detection published in ACS Photonics at the beginning of 2016. That work has many similar properties and should be published. Also, there is a work that discusses the trapping of quantum dots and the Kramers hopping hypothesis that is not mentioned in Lab on Chip 2013 (DOI:10.1039/C3LC00003F). I also noticed one of the references was repeated.

2) The fluctuations of the second harmonic may also be explained by orientation of the quantum dots. As they rotate, the SHG tensor should change? How do the authors rule out this consideration?

3) On page 10, the authors say that the polarization was along the x-direction. Did they mean y, as the simulations would suggest?

4) Did the authors observe any 3 photon fluorescence? (This would rule out the rotation hypothesis since it is not linked to inversion symmetry).

Reviewer #2 (Remarks to the Author):

First I want to mention that I found the work described in the manuscript interesting. However, I think there are some aspects that should be clarified and improved before publishing. These are the following:

1.- The authors describe their method as “material-free, label-free, high contrast and low power”. I think they should remove the concept of “material-free”, which I assume to be an abuse of speech. Clearly to perform the second harmonic generation the system requires several materials such as a plasmonic structure to confine and enhance the electromagnetic fields, collection optics, etc.

Furthermore, they could reconsider using the concept of low power and substitute it by “low Intensity”, as in the end the authors quantify and study the effects of E^2. While one can directly relate the Intensity with E^2, additional information on the geometry is needed to relate E^2 with the power.

2.- In line 51, I would change the article “uncomfortable”, which expresses a highly subjective opinion rather than an actual technical difficulty.

3.- In lines 58-60 the authors mention several position detection schemes for nanoparticles. They

make a choice of sophisticated methods but forget to mention the simplest and most commonly used ones, which are the ones their method should compare to.

Direct imaging of the dipole scatterer and fitting its centroid (see for example : BRZOBOHATÝ, Oto, et al. Three-dimensional optical trapping of a plasmonic nanoparticle using low numerical aperture optical tweezers. Scientific reports, 2015, vol. 5, p. 8106.)

Back-focal plane interferometry which is extensively used in optical tweezers (see for a simple model of the method Gittes, F., & Schmidt, C. F. (1998). Interference model for back-focal-plane displacement detection in optical tweezers. Optics letters, 23(1), 7-9.)

4.- In figure 1(b) the colorbar for the scale in the electric field lacks units. Same for figure 3(b).

5.- I like how the authors consider the effect of the fs-laser (detection) regarding trapping. However, I miss a control experiment where they show how actually similar data with their experiment cannot be obtained by just looking at the trapping laser. In other words, repeat what they show in plots 5(b) but without their detection laser. I think this is important in order to sustain their claim that using a second harmonic generated signal provides more advantages than just monitoring the transmitted light of the trapping laser.

6.- The authors identify the existence of a krammers hopping process from the spikes observed in the SH signal during a trapping interval and the Lorentzian shape of the computed Power Spectral density (PSD) . I do agree they both seem to point towards such effect, but the authors should extend a bit further their analysis and discussion. More specifically regarding the shape of the PSD, and the power dependence of the cutoff frequency.
The reason is that an overdamped-lorentzian shaped Lorentzian PSD appears also for any trapped particle in a harmonic potential (not need of a double well). This has been recently shown in a plasmonic trapping experiment with nearly identical configuration (see: Mestres et al. Unraveling the optomechanical nature of plasmonic trapping. Light: Science & Applications, 5(7), 2016)
Therefore in order to strengthen the “krammer´s hopping process” claim, the authors should clarify how their data supports the “krammers hopping” instead of a “diffusion in a harmonic potential” despite the PSD looking qualitatively so similar.

---------------------------------Reviewer #1 (Remarks to the Author):-------------------------------

In the manuscript "Non-fluorescent nanoscopic monitoring of a single trapped nanoparticle via nonlinear point sources" the authors use CW and fs lasers to trap quantum dots with nanoapertures in gold film. They monitor the trapping by changes to the fundamental and second harmonic. The second harmonic shows greater sensitivity to nearfield intensity and therefore, they suggest that the fluctuations observed are from Kramers hopping. Overall, I think that the manuscript presents remarkable trapping results with substantial SHG from a single 4 nm QD. I am not entirely convinced of the Kramers hopping hypothesis, but I do not feel this should preclude publication.

- Thank you very much for reviewing our manuscript and providing constructive remarks. We have attempted to address all the points carefully, and hope you find each point adequately treated.

- In addition, we have added one section (Section 6) in the supplementary information for more specific analysis and discussion regarding the Kramers hopping.

1) I note that the authors have not carefully reviewed the literature. For example, there is a work on trapping of quantum dots and two photon fluorescence detection published in ACS Photonics at the beginning of 2016. That work has many similar properties and should be published. Also, there is a work that discusses the trapping of quantum dots and the Kramers hopping hypothesis that is not mentioned in Lab on Chip 2013 (DOI:10.1039/ C3LC00003F). I also noticed one of the references was repeated.

- We thank the reviewer for pointing this out.

- We have added the following references;
[21] Marin, B. C., Hsu, S.-W., Chen, L., Lo, A., Zwissler, D. W., Liu, Z. & Tao, A. R. Plasmon-enhanced two-photon absorption in photoluminescent semiconductor nanocrystals, ACS Photonics ***3****, 526-531 (2016).*
[22] Zehtabi-Oskuie, A., Jiang, H., Cyr, B. R., Rennehan, D. W., Al-Balushi, A. A. & Gordon, R. Double nanohole optical trapping: dynamics and protein-antibody co-trapping. Lab Chip. ***13****, 2563-2568 (2013).*

- And, we have removed the duplicate references of "*[18] Kotnala, A. & Gordon, R. Quantification of high-efficiency trapping of nanoparticles in a double nanohole optical tweezer. Nano Lett. 14, 853–856 (2014).*" and "*[19] Berthelot, J., Aćimović, S. S., Juan, M. L., Kreuzer, M. P., Renger, J. & Quidant, R. Three-dimensional manipulation with scanning near-field optical nanotweezers. Nat. Nanotechnol. 9, 295–299 (2014).*"

- The reference numbers have been adjusted to match the content of the text.

2) The fluctuations of the second harmonic may also be explained by orientation of the quantum dots. As they rotate, the SHG tensor should change? How do the authors rule out this consideration?

- Yes, the SH intensity could fluctuate by the rotation of the QD with change of the SHG tensor.

- However, we found in experiment that the fundamental (I_{ω}) and second-harmonic ($I_{2\omega}$) signals are almost perfectly synchronized with each other, as shown in below Fig. R1 or Fig. S6 of the supplementary information.

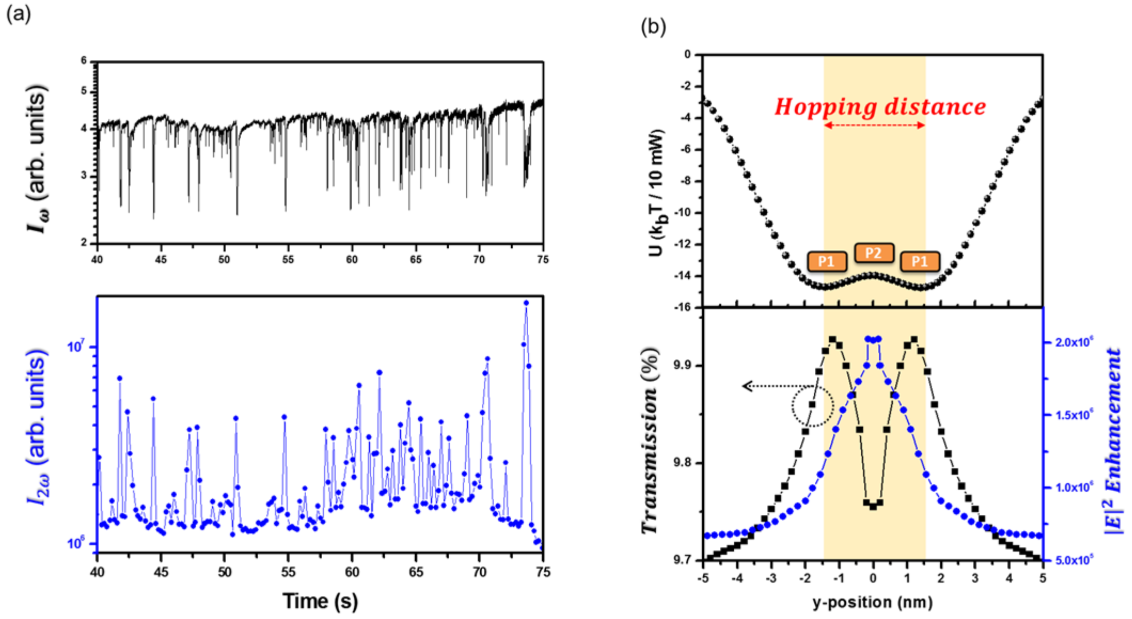

Fig. R1. (a) Measured fundamental (I_{ω}) and second-harmonic ($I_{2\omega}$) signals when P_{cw} = 40 mW and P_{fs} = 4.0 mW. Here, the integration times for I_{ω} and $I_{2\omega}$ detection are 2 ms and 200 ms, respectively. (b) Calculated optical potential, transmission, and $|E|^2$ enhancement when a 4.4-nm QD moves along the *y*-direction.

1. Note that the transmission of the fundamental signal (I_{ω}) depends mainly on the QD position in the antenna, independent of the rotational motion.
2. The rotational frequency of the 5-nm QD at room temperature is calculated to be ~1.1 MHz, much larger than a few Hz observed in our experiment.
   [Jinda Lin et al. Optical trapping and rotation of airborne absorbing particles with a single focused laser beam, Appl. Phys. Lett. 104, 101909 (2014)]

Based on these observations, we have concluded that the $I_{2\omega}$ fluctuation due mainly to the translational movement of the QD.

- We have added the following sentence in line 19 at page 12:
  "Additionally, in the transmitted fundamental signal (I_{ω}), similar negative spikes are simultaneously observed mainly due to the translational movement of the trapped QD, as shown in Fig. 5(d) [see the Supplementary Info.]"

- And, we have added Fig. R1 in Section 6 (as Fig. S8) of the supplementary information.

3) On page 10, the authors say that the polarization was along the x-direction. Did they mean y, as the simulations would suggest?

- We thank the reviewer for pointing out our mistakes. We have corrected "*x-direction*" in page 10 and "*x-axis*" in page 17 to "*y-direction*" and "*y-axis*", respectively.

4) Did the authors observe any 3 photon fluorescence? (This would rule out the rotation hypothesis since it is not linked to inversion symmetry).

- We have not observed any 3 photon fluorescence in our experiment due to its weak intensity. As mentioned in the answer of comment-2, we could rule out the rotation hypothesis in our experiment using the information of synchronization between the fundamental and SH signals.

---------------------------------Reviewer #2 (Remarks to the Author):---------------------------------

First I want to mention that I found the work described in the manuscript interesting. However, I think there are some aspects that should be clarified and improved before publishing. These are the following:

- Thank you very much for reviewing our manuscript and providing constructive remarks. We have attempted to address all the points carefully, and hope you find each point adequately treated.

1) The authors describe their method as "material-free, label-free, high contrast and low power". I think they should remove the concept of "material-free", which I assume to be an abuse of speech. Clearly to perform the second harmonic generation the system requires several materials such as a plasmonic structure to confine and enhance the electromagnetic fields, collection optics, etc.

- We have removed the phrase of "*material-free*" in Abstract and Summary.

Furthermore, they could reconsider using the concept of low power and substitute it by "low Intensity", as in the end the authors quantify and study the effects of E^2. While one can directly relate the Intensity with E^2, additional information on the geometry is needed to relate E^2 with the power.

- We agree with the reviewer that the concept of optical intensity rather than the concept of optical power can more accurately represent the property of our proposed scheme.

- So, we have modified the following sentences;
  (Abstract, line 5) "*... with low power of a 1,560-nm ...*" → "*... with low intensity of a 1,560-nm ...*"
  (Abstract, line 10) "*The material-free, label-free, high-contrast, and low-power characteristics of the proposed scheme…* " → "*The label-free, high-contrast, and low-intensity operation characteristics of the proposed scheme…*"

- The information on the geometry can be found in line 1 at page 10:
  "The beam diameter focused through a 60× objective lens (numerical aperture = 0.65) is measured to be 2.0 μm"

- In section of "Experiment", we have added the following laser intensities;
  (Page 10, line 10) "*With a low fs-laser power of 4.0 mW,…*" → "*With a low fs-laser power of 4.0 mW (intensity $I_{fs} = P_{fs}/A_{beam} = 0.12$ MW/cm^2, where A_{beam} is the area of the focused beam),…*"
  (Page 10, line 14) "*When P_{fs} is increased to 5.5 mW, …*" → "*When P_{fs} is increased to 5.5 mW ($I_{fs} = 0.18$ MW/cm^2), …*"
  (Page 10, line 16) "*If P_{fs} is further increased to 7.5 mW, …*" → "*If P_{fs} is further increased to 7.5 mW ($I_{fs} = 0.24$ MW/cm^2), …*"
  (Page 12, line 5) "*The CW trapping laser power of 10 mW is …*" → "*The CW trapping laser power of 10 mW ($I_{cw} = 0.32$ MW/cm^2) is …*"
  (Page 12, line 8) "*When the trapping laser power P_{cw} is increased to 40 mW, …*" → "*When the trapping laser power P_{cw} is increased to 40 mW ($I_{cw} = 1.27$ MW/cm^2), …*"

- In section of "Summary", we have modified the following sentence;

(Page 14, line 8) "… *with low optical power on the order of 1 mW.*" → "*… with low optical intensity less than 0.4 MW/cm^2.*"

- The $|E_{in}|^2$ and intensity (I) of an incident beam follow the relation of $I = \frac{cn\varepsilon_0}{2}|E_{in}|^2$. And, this $|E_{in}|^2$ is extremely enhanced through the 3-D tapered 5-nm-gap plasmonic nanoantenna by a factor (enhancement factor = $|E_{max}|^2$ / $|E_{in}|^2$) of 6.1 × 10^5.

- As described in the manuscript, the large field enhancement in a local space via the plasmonic nanoantenna leads to a large field gradient, which results in a large optical force to the nanoparticle.

- We have added one section (Section 2, shown below) in the supplementary information to provide the additional information of $|E|^2$ enhancement ($|E_{max}|^2$ / $|E_{in}|^2$) as a function of geometry of the plasmonic nanoantenna.

==

2. Field enhancement in plasmonic nanoantenna

The mode volume and $|E|^2$ enhancement of the 3D gap-plasmon antenna are plotted as a function of g in Fig. S2. Here, θ is fixed at 65°, and the length and width of the antenna are 200 and 160, respectively. The pump beam (beam diameter = 2.0 μm) is illuminated from the SiO_2 substrate side. The $|E|^2$ enhancement is defined as the ratio between the maximum $|E|^2$ values of the antenna mode and the incident wave. Photon confinement is clearly noticeable when g is less than 20 nm. When g is reduced from 50 nm to 2 nm, the mode volume decreases from 4.0 × 10^{-5} λ^3 to 4.4 × 10^{-9} λ^3 and the $|E|^2$ enhancement increases from 1.5 × 10^4 to 2.0 × 10^6. For an antenna with g = 5 nm, the mode volume and $|E|^2$ enhancement are calculated to be 6.1 × 10^{-8} λ^3 and 6.1 × 10^5, respectively. The $|E|^2$ enhancement is dependent on the mode volume, coupling efficiency, and joule losses. Here, the coupling efficiency is estimated to be 20%, and the ratio of the metal absorption among the total loss from the antenna is 25%.

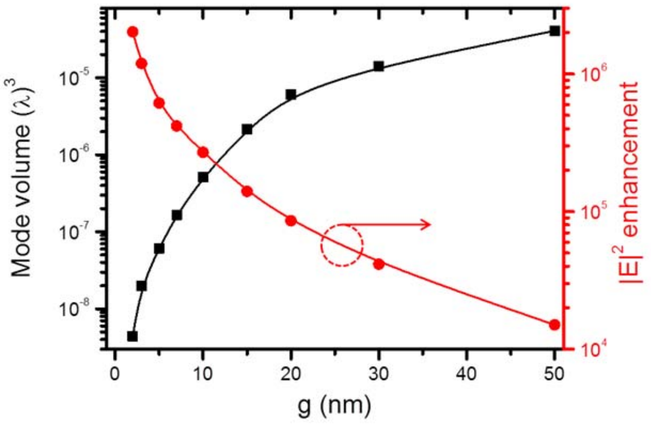

Fig. S2. Mode volume and $|E|^2$ enhancement as a function of g at a fixed θ of 65°. When g = 5 nm, the mode volume and $|E|^2$ enhancement are calculated to be 6.1 × 10^{-8} λ^3 and 6.1 × 10^5, respectively. Here, all the simulation data were taken at resonant wavelengths.

2) In line 51, I would change the article "uncomfortable", which expresses a highly subjective opinion rather than an actual technical difficulty.

- We have modified the phrase of "*uncomfortable pre-process of attaching …*" in page 2 as "*additional pre-process of attaching …*".

3) In lines 58-60 the authors mention several position detection schemes for nanoparticles. They make a choice of sophisticated methods but forget to mention the simplest and most commonly used ones, which are the ones their method should compare to.

Direct imaging of the dipole scatterer and fitting its centroid (see for example : BRZOBOHATÝ, Oto, et al. Three-dimensional optical trapping of a plasmonic nanoparticle using low numerical aperture optical tweezers. Scientific reports, 2015, vol. 5, p. 8106.)

Back-focal plane interferometry which is extensively used in optical tweezers (see for a simple model of the method Gittes, F., & Schmidt, C. F. (1998). Interference model for back-focal-plane displacement detection in optical tweezers. Optics letters, 23(1), 7-9.)

- We thank the reviewer for suggesting the references regarding the detection of nanoparticles. We have added the following references;
  [11] Brzobohatý, O., Šiler, M., Trojek, J., Chvátal, L., Karásek, V., Paták, A, Pokorná. Z., Mika, F. & Zemánek, P. Three-dimensional optical trapping of a plasmonic nanoparticle using low numerical aperture optical tweezers. Sci. Rep. ***5****, 8106 (2015).*
  [12] Gittes, F. & Schmidt, C. F. Interference model for back-focal-plane displacement detection in optical tweezers. Opt. Lett. ***23****, 7-9 (1998).*

- And, we have modified the sentence in line 15 at page 3;
  (Page 3, line 15) "*… , such as high-precision surface-enhanced Raman spectroscopy (SERS) [7, 8], the cavity-enhanced detection methodology [9], and interferometric scattering microscopy (iSCAT) [10].*" → "*… , such as high-precision surface-enhanced Raman spectroscopy (SERS) [7, 8], cavity-enhanced detection methodology [9], interferometric scattering microscopy (iSCAT) [10], centroid fitting methodology [11], and back-focal-plane interferometry [12].*"

- The reference numbers have been adjusted to match the content of the text.

4) In figure 1(b) the colorbar for the scale in the electric field lacks units. Same for figure 3(b).

- We thank the reviewer for pointing this out. We have added the scales of color-bars in Figs. 1(b) and 3(b) of the main text.

5) I like how the authors consider the effect of the fs-laser (detection) regarding trapping. However, I miss a control experiment where they show how actually similar data with their experiment cannot be obtained by just looking at the trapping laser. In other words, repeat what they show in plots 5(b) but without their detection laser. I think this is important in order to sustain their claim that using a second harmonic generated signal provides more advantages than just monitoring the transmitted light of the trapping laser.

- We thank the reviewer for pointing this out. We have added the control experimental result for P_{cw} = ON/OFF & P_{fs} = OFF in Fig. S7 (below figure) of the supplementary information.

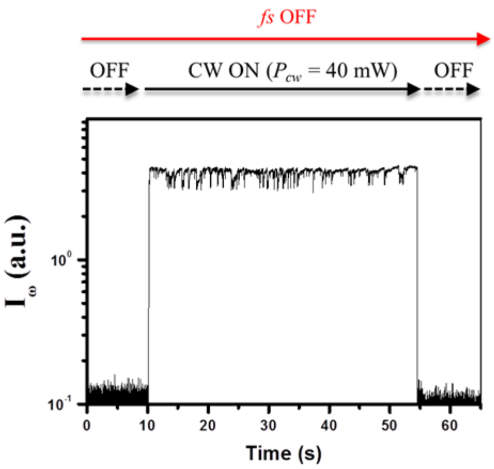

Fig. S7. Time trace of the measured transmitted fundamental-signal intensity (I_ω) with QDs. Here, the CW laser (P_{cw} = 40 mW) is turned ON and OFF while the *fs*-laser is turned OFF.

- And, we have added the following sentence in Section 5 of the supplementary information; "*Figure S7 shows the control experiment result when the trapping CW laser is turned ON and OFF while the detection fs-laser is OFF.*"

- Figure 5 of the manuscript (below figure) already represents that the monitoring of a background-free SH signal ($I_{2\omega}$) provides more advantage than just monitoring a transmitted light (I_ω) of the trapping laser. Looking at the trapped moment (*t*=15*s*) of the QD, monitoring the I_ω signal makes it difficult to distinguish whether the QD is trapped, due to a sudden increase in the I_ω signal when the trapping CW laser is turned on. On the other hand, the background-free $I_{2\omega}$ signal clearly distinguishes whether the QD is trapped or not and even shows the dynamics of the trapped QD.

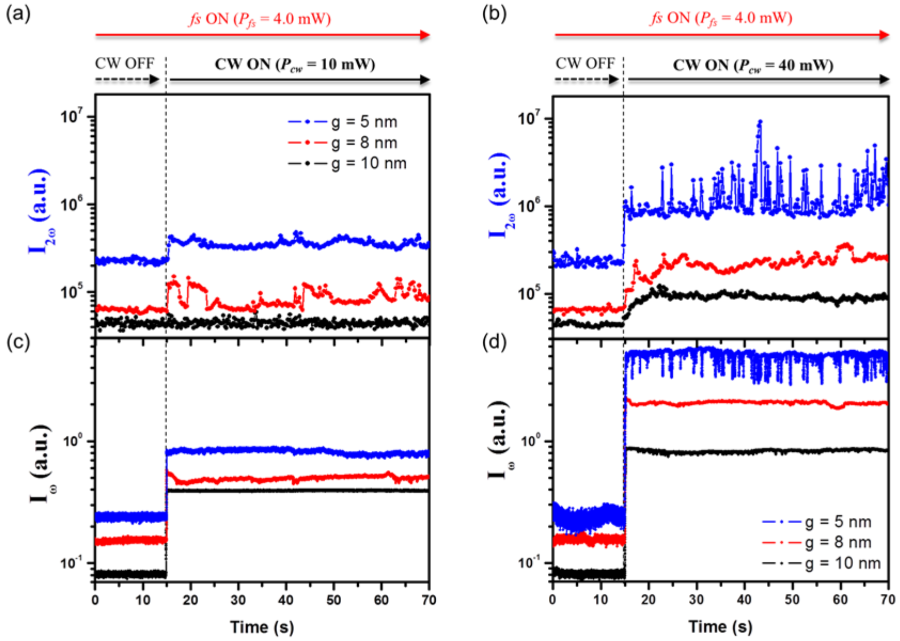

Fig. 5. (a, b) SH intensities ($I_{2\omega}$). (c, d) Transmitted fundamental wave intensities (I_ω). The CW laser with different powers of 10 and 40 mW is turned ON at t = 15 s. The 4.0 mW *fs*-laser remains ON.

- To further emphasize the advantage of monitoring the SH signals in the manuscript, we have added the following sentence in line 1 at page 12 of the main text;
  (Page 12, line 1) "*Looking at the trapped moment (t = 15s) of the QD, monitoring the I_ω signal makes it difficult to distinguish whether the QD is trapped, due to a sudden increase in the I_ω signal when the trapping CW laser is turned on. On the other hand, the $I_{2\omega}$ signal clearly tells us whether the QD is trapped or not and even shows the dynamics of the trapped QD.*"

6) The authors identify the existence of a Kramers hopping process from the spikes observed in the SH signal during a trapping interval and the Lorentzian shape of the computed Power Spectral density (PSD). I do agree they both seem to point towards such effect, but the authors should extend a bit further their analysis and discussion. More specifically regarding the shape of the PSD, and the power dependence of the cutoff frequency.

The reason is that an overdamped-lorentzian shaped Lorentzian PSD appears also for any trapped particle in a harmonic potential (not need of a double well). This has been recently shown in a plasmonic trapping experiment with nearly identical configuration (see: Mestres et al. Unraveling the optomechanical nature of plasmonic trapping. Light: Science & Applications, 5(7), 2016)

Therefore in order to strengthen the "Kramers hopping process" claim, the authors should clarify how their data supports the "Kramers hopping" instead of a "diffusion in a harmonic potential" despite the PSD looking qualitatively so similar.

- We thank the reviewer for pointing this out.

- We have added one section (Section 6, described below) in the supplementary information to describe more specific analysis and discussion regarding "Kramers hopping". This section clarifies that our PSD data supports the "Kramers hopping" instead of a "diffusion in a harmonic potential" by analyzing the shape of the PSD and the power dependency on the roll-off frequency

==

6. Kramers Hopping

The fundamental (I_ω) and second-harmonic ($I_{2\omega}$) spike signals are observed when a 40-mW CW laser and a 4-mW fs-laser are pumped, as shown in Fig. S8(a). And, both signals are synchronized with each other, indicating that the $I_{2\omega}$ fluctuation is mainly due to the translational movement of the trapped QD. We attribute this phenomenon to the Kramers hopping associated with the double potential well formed near the two hot plasmonic points, as shown in Fig. S8(b). The low-frequency characteristics of these spikes support our interpretation. That is, the QD hops back and forth between two shallow potential wells randomly at a low frequency. It generates negative I_ω and positive $I_{2\omega}$ spikes at the

instant that the QD passes by the barrier, due to the sudden decrease of transmittance and increase of $|E|^2$ enhancement, respectively, as shown in Fig. S8(b)

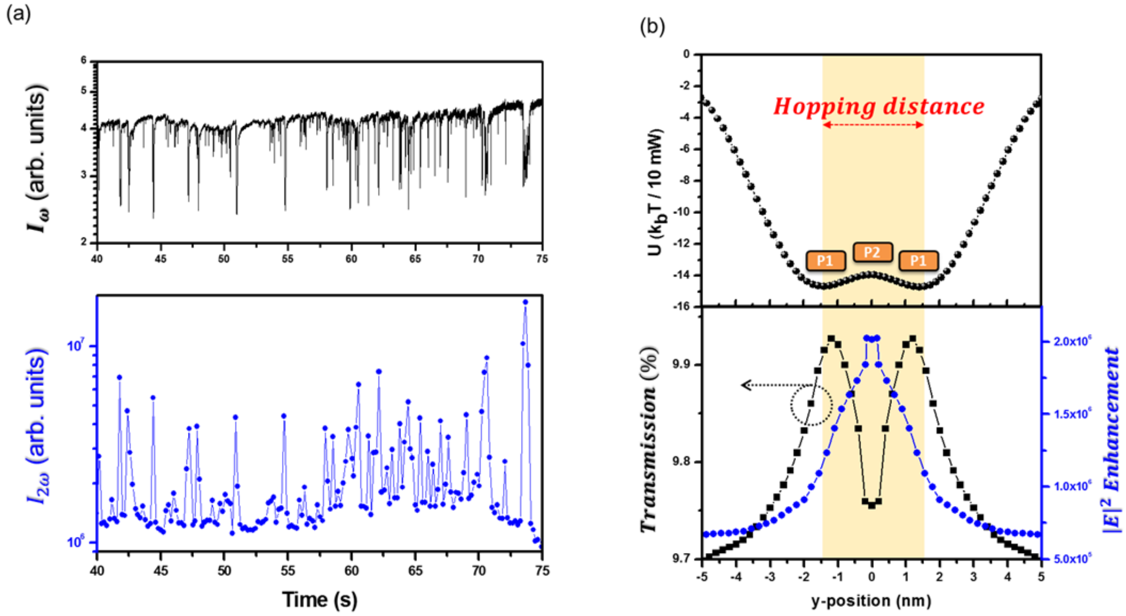

Fig. S8. (a) Measured fundamental (I_ω) and second-harmonic ($I_{2\omega}$) signals under the condition of P_{cw} = 40 mW and P_{fs} = 4.0 mW. Here, the integration times for I_ω and $I_{2\omega}$ detection are 2 ms and 200 ms, respectively. (b) Calculated optical potential, transmission, and $|E|^2$ enhancement when a 4.4-nm QD moves along the y-direction.

The hopping rate is analysed by using the power spectrum density (PSD) method, as shown in Figs. 6(c) and 6(d) of the main text. The roll-off frequency (corner frequency) of the Kramers hopping is estimated to be 3.0 and 0.5 Hz for P_{cw} of 40 and 30 mW, respectively. In order to clarify that our PSD data supports the "Krammrs hopping" instead of a "diffusion in a harmonic potential", we estimate the roll-off frequency of oscillation, assuming that a QD is trapped in a single harmonic potential [4 - 7]. In this case, the roll-off frequency is given by $f_0 = \alpha(2\pi\beta)^{-1}$, where α is the trap stiffness, β is the drag coefficient ($\beta = 6\pi\eta r$), r is the particle radius, and η is the viscosity of the water. And, the trapping stiffness α can be calculated using the equipartition principle of $\alpha\langle x^2\rangle = k_bT$. Here, we set x = 5 nm, because the potential width is comparable to the size of the antenna gap. Using those parameters, the roll-off frequency is estimated to be $f_0 \sim 6\times10^5$ Hz, which is significantly larger than the frequency (a few Hz) obtained from our experiment. So, the assumption that the particle oscillates simply in a single harmonic potential well can be ruled out.

We additionally tested the power dependence on the roll-off frequency, as shown in Fig. S9, as increasing the trapping CW laser power (P_{cw}) from 10 to 40 mW. We can see that the roll-off frequency increases with the pumping power, where the roll-off frequency is

in the range of 0 ~ 3 Hz. This observation indicates that the trapped QD is in the underdamped state of the Kramers hopping [8 - 10].

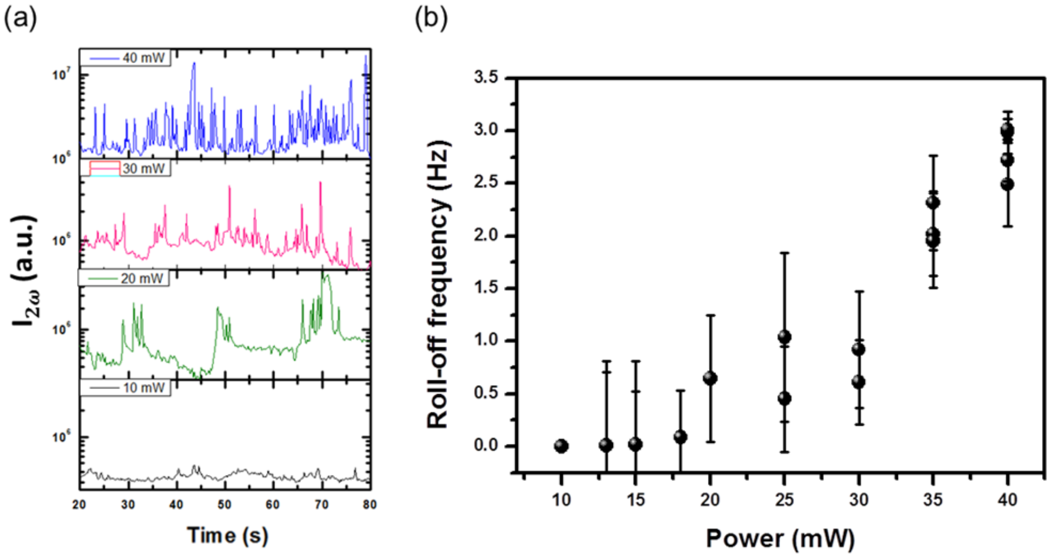

Fig. S9. (a) Measured SH signals ($I_{2\omega}$) with CW laser powers P_{cw} of 40, 30, 20 and 10 mW. (b) Measured roll-off frequency as a function of P_{cw}.

REVIEWERS' COMMENTS:

Reviewer #1 (Remarks to the Author):

The authors have sufficiently addressed my concerns. I recommend publication.

Reviewer #2 (Remarks to the Author):

I am satisfied with the changes implemented by the authors. I would recommend the article for publication.

REVIEWERS' COMMENTS:

Reviewer #1 (Remarks to the Author):
The authors have sufficiently addressed my concerns. I recommend publication.

Reviewer #2 (Remarks to the Author):
I am satisfied with the changes implemented by the authors. I would recommend the article for publication.

- We thank the reviewers for their valuable comments and suggestions.
